# Genetic diversity and virulence profiles of *Listeria monocytogenes* recovered from bulk tank milk, milk filters, and milking equipment from dairies in the United States (2002 to 2014)

**DOI:** 10.1371/journal.pone.0197053

**Published:** 2018-05-09

**Authors:** Seon Woo Kim, Julie Haendiges, Eric N. Keller, Robert Myers, Alexander Kim, Jason E. Lombard, Jeffrey S. Karns, Jo Ann S. Van Kessel, Bradd J. Haley

**Affiliations:** 1 Environmental Microbial and Food Safety Laboratory, Beltsville Agricultural Research Center, Agricultural Research Services, United States Department of Agriculture, Beltsville, MD, United States of America; 2 Maryland Department of Health and Mental Hygiene, Baltimore, MD, United States of America; 3 Center for Epidemiology and Animal Health, USDA-Animal and Plant Health Inspection Service, Veterinary Services, Fort Collins, CO, United States of America; Gaziosmanpasa University, TURKEY

## Abstract

Unpasteurized dairy products are known to occasionally harbor *Listeria monocytogenes* and have been implicated in recent listeriosis outbreaks and numerous sporadic cases of listeriosis. However, the diversity and virulence profiles of *L*. *monocytogenes* isolates recovered from these products have not been fully described. Here we report a genomic analysis of 121 *L*. *monocytogenes* isolates recovered from milk, milk filters, and milking equipment collected from bovine dairy farms in 19 states over a 12-year period. In a multi-virulence-locus sequence typing (MVLST) analysis, 59 Virulence Types (VT) were identified, of which 25% were Epidemic Clones I, II, V, VI, VII, VIII, IX, or X, and 31 were novel VT. In a multi-locus sequence typing (MLST) analysis, 60 Sequence Types (ST) of 56 Clonal Complexes (CC) were identified. Within lineage I, CC5 and CC1 were among the most abundant, and within lineage II, CC7 and CC37 were the most abundant. Multiple CCs previously associated with central nervous system and maternal-neonatal infections were identified. A genomic analysis identified variable distribution of virulence markers, *Listeria* pathogenicity islands (LIPI) -1, -3, and -4, and stress survival island-1 (SSI-1). Of these, 14 virulence markers, including LIPI-3 and -4 were more frequently detected in one lineage (I or II) than the other. LIPI-3 and LIPI-4 were identified in 68% and 28% of lineage I CCs, respectively. Results of this analysis indicate that there is a high level of genetic diversity among the *L*. *monocytogenes* present in bulk tank milk in the United States with some strains being more frequently detected than others, and some being similar to those that have been isolated from previous non-dairy related outbreaks. Results of this study also demonstrate significant number of strains isolated from dairy farms encode virulence markers associated with severe human disease.

## Introduction

*Listeria monocytogenes* is an environmentally-adapted Gram-positive foodborne pathogen occasionally causing disease in humans and other mammals. It is the primary causative agent of listeriosis, with manifestations of disease in humans that can range from mild illness such as gastroenteritis in healthy individuals to severe infections of the central nervous system (CNS) such as meningitis, meningoencephalitis, cerebral abscesses, cerebritis, and bacteremia in immunocompromised, elderly, and young individuals. Although symptomatic infections may be relatively rare, the case fatality rate ranges from 20% to 50% in immunocompromised individuals with disseminated infections [1]. In pregnant women listeriosis is typically manifested as a mild illness or an asymptomatic infection, but pregnant women are 10 to 24 times more likely to develop listeriosis than the general population [2–5]. The most severe cases occur in perinates and neonates with the case fatality rate ranging from 20% to 45% [6, 7] in this group.

Recent studies have demonstrated that certain strains are relatively hypervirulent and are capable of causing severe central nervous system infections in immunocompromised individuals as well as perinatal infections [4, 8, 9]. The severity of illness as well as the manifestations of infections may be in part due to strain-specific genomic features. For example, truncated *inlA* (coding for internalin A which plays a major role in intestinal cell invasion) is associated with a marked attenuation in virulence, and strains encoding this truncation are frequently identified in the food supply [10]. However, the full-length *inlA* gene, and *Listeria* pathogenicity islands (LIPI) -3 and -4 are often identified in strains that are the causative agents of severe disease which are also relatively infrequently found in foods [9]. Further, recent studies demonstrated that some strains are more likely to be isolated from certain environments, materials or foods than others [9, 11–14]. These data indicate that *L*. *monocytogenes* is highly heterogenous and its distribution in food products and the environment may be non-random. However, analyses of the genomic diversity of *L*. *monocytogenes* have frequently focused on clinical isolates rather than foodborne or environmental isolates.

Raw (unpasteurized) milk represents a potential source of foodborne infections globally and studies have shown this product to be contaminated with human pathogens such as *L*. *monocytogenes*, *Salmonella enterica*, *Campylobacter* spp., *Yersinia enterocolitica*, and enterohemorrhagic *Escherichia coli* [15–19]. Typically, these pathogens and other bacteria are reduced in milk during pasteurization, a process of pathogen abatement that has, at least in part, been a significant factor in the reduction of infant and childhood mortality rates since the late-19^th^ Century [20, 21]. However, consumption of raw milk is gaining popularity and some states have legalized the intra-state sale of this product to the public. Several notable disease outbreaks have been linked to raw milk consumption in the United States and abroad and an analysis by Mungai et al. [22] demonstrated an increase in disease outbreaks in recent years due to the consumption of unpasteurized milk [23–26].

Bulk tank milk (BTM) refers to chilled, raw milk that is stored in bulk prior to collection by a milk hauler. As this milk is an aggregate of the herd, bacteriological tests can be conducted to evaluate the presence of human and bovine pathogens within the herd. The presence of pathogens in BTM and bulk tank milk filters (BTMF) is typically due to fecal-contamination during the milking process, pathogen shedding from the udder, and/or dispersal of cells from biofilms established in milking equipment [16, 27]. The presence of *L*. *monocytogenes* in BTM has been attributed to fecal contamination of milk by cows fed contaminated and improperly stored silage [28–30] and recent surveys have reported the repeated detection of *L*. *monocytogenes* in BTM, feces, and environmental samples collected from dairy farms [16, 17, 31–38]. However, the prevalence of *L*. *monocytogenes* contamination of BTM and BTMF in the United States is relatively low [16, 17]. Of the studies in which serological testing was conducted, serogroups 1/2a and 1/2b were identified as the most prevalent of the major serogroups among isolates collected from BTM [16, 17]. However, information regarding the molecular diversity at a finer scale than the serogroup classification is currently lacking. As stated earlier, recent studies have demonstrated a high level of heterogeneity within this species with some strains being putatively avirulent and others being more strongly associated with severe CNS and MN infections [9–10, 33]. These health outcomes have been linked to polymorphisms in known virulence genes and the presence of pathogenicity islands [9–10, 33]. Thus, the potential association of specific strains with clinical infections makes it essential to further characterize strains present in food products.

The aim of this study was to evaluate the genomic diversity and virulence profiles of isolates of both of the major *L*. *monocytogenes* lineages (I and II) collected from dairies in major dairy producing states in the United States. To accomplish this, we determined the phylogeny, sequence types (ST), clonal complexes (CC), virulence types (VT), and the distribution of virulence markers and pathogenicity islands among isolates collected from BTM, BTMF, and milking equipment in US dairy operations in 19 states over a 12-year period.

## Materials and methods

Confirmed *L*. *monocytogenes* isolates were selected from previous National Animal Health Monitoring System (NAHMS) dairy surveys conducted in 2002, 2007, and 2014, as well as routine dairy farm sampling conducted on a single commercial dairy farm in New York State in 2004, 2005, and 2007 [16, 17, 38, 39]. The states were selected as major dairy producing states by NAHMS. To adequately investigate the diversity within *L*. *monocytogenes* lineages I and II, an attempt was made to select similar numbers of lineage I and II isolates based on results of PCR-serogrouping of the isolates. Thus, results of the analyses for each lineage are discussed separately.

For this study, 76 of the selected isolates were recovered from BTM, 43 from BTMF, and 2 from milking equipment. Isolates from BTMF were collected after 2002. All isolates used in this study were previously confirmed as *L*. *monocytogenes*, and the serogroups were determined following the methods of Doumith et al [40]. DNA was extracted using the Qiagen DNeasy kit (Qiagen, Hilden, Germany). Genomic DNA sequencing libraries were prepared using the Nextera XT Library Preparation Kits (Illumina, La Jolla, CA). *L*. *monocytogenes* genomes were sequenced by using 250-bp paired-end libraries, with a MiSeq reagent kit (v2), on a MiSeq system (Illumina), per the manufacturer's instructions. Raw sequencing reads were deposited in the NCBI SRA database under BioProject PRJNA215355 (*Listeria monocytogenes* database, US Food and Drug Administration GenomeTrakr Project) (S1 Table). Sequencing contaminants were trimmed and removed using Trimmomatic and Deconseq [41, 42]. The *L*. *monocytogenes* genomes were assembled using SPAdes V. 3.8.0 [43]. Contigs < 500 bp were trimmed from each genome file. ST, CC, deduced serogroups, and lineage assignments were determined *in silico* using the *L*. *monocytogenes* MLST webserver hosted by the Pasteur Institute [44] and VT were determined *in silico* using the scheme described by Zhang et al. [45]. Novel ST and VT assignments are available at the website for each typing database [44, 46]. Presence of pathogenicity islands and virulence genes [9, 47] were identified using BLASTN with e values set to a minimum of 1x10^-30^, a minimum percent identity set to 90%, and a minimum query coverage of 80%. Differences in the proportions of virulence factors and pathogenicity islands identified in lineages I and II were evaluated, after duplicate CCs were removed, by conducting a Fisher’s exact test in R (https://cran.r-project.org). Differences in the proportions of virulence markers between lineages were considered significant when P < 0.05.

Single nucleotide polymorphisms (SNPs) were identified among all genomes using Parsnp with parameters set to default with a recombination filter (-x), all genomes included (-c), and random reference genome selection (-r!) which chose genome ARS-CC9338 [48]. A Maximum Likelihood phylogenetic tree was inferred with 100 bootstrap replicates using RAxML v. 8.0 with parameters set to default [49].

Acquired antibiotic resistance genes were identified using the ResFinder (v. 1.0) program hosted by the Center for Genomic Epidemiology webserver [50, 51]. The functionality of resistance genes identified *in silico* was evaluated using an automated microdilution procedure (Sensititre™, ThermoFisher, Lenexa, KS) and the Sensititre™ Gram Positive MIC Plate. Antimicrobial minimum inhibitory concentrations (MICs) breakpoints were based upon epidemiological cut-off values (ECOFFs) set by the European Committee on Antimicrobial Susceptibility Testing (EUCAST).

## Results

**Fig 1:**
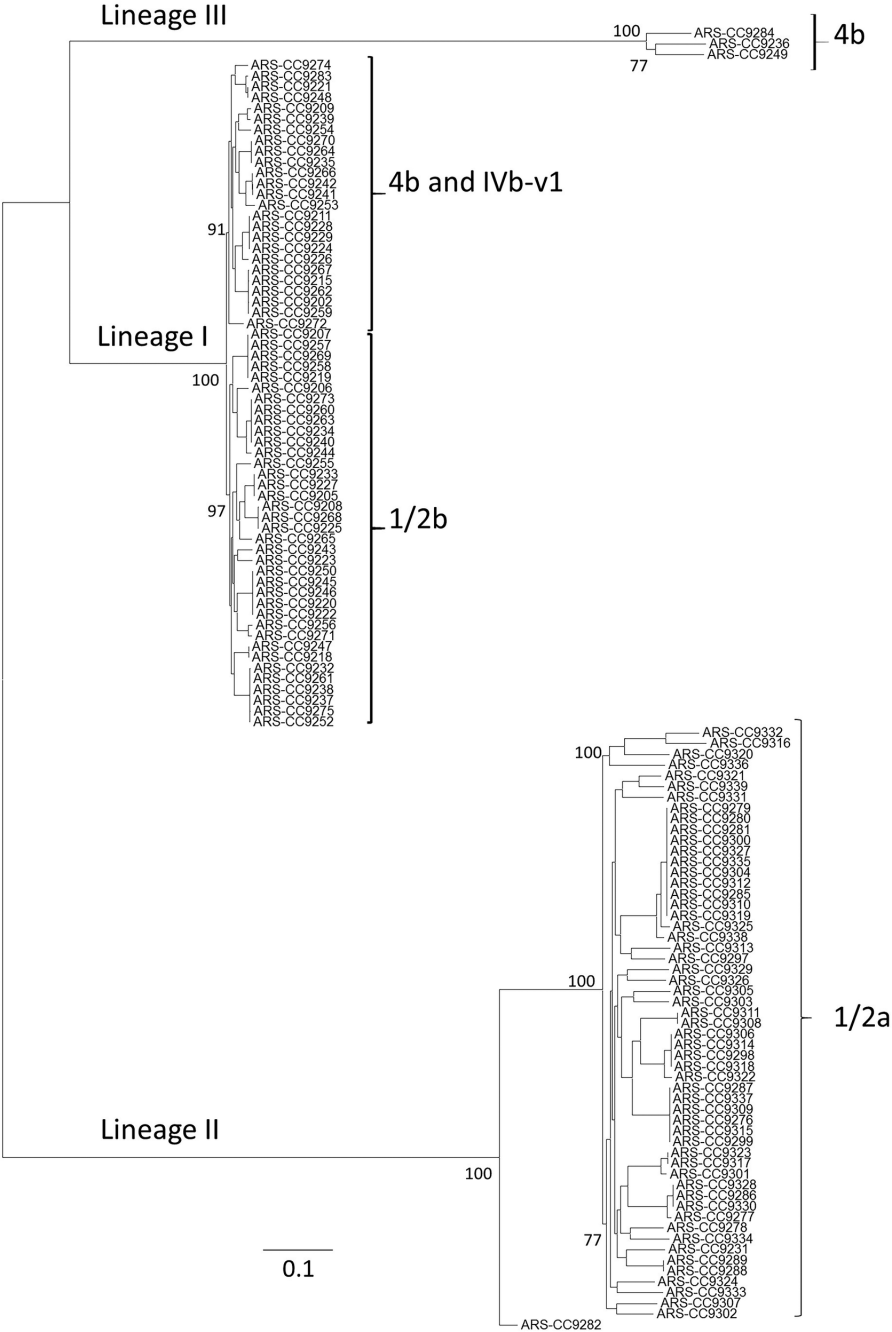
Maximum likelihood phylogenetic tree of 121 *L*. *monocytogenes* isolates collected from US dairies. The displayed phylogeentic tree is based on 166,603 core-genome SNPs identified among the study isolates. The phylogenetic tree was inferred using the General Time Reversible model of nucleotide substitution in RAxML with 100 bootstrap replicates. Bar length represents number of substitutions per site.

The genomes 121 *L*. *monocytogenes* isolates recovered from BTM, BTMF, and milking equipment [16, 17, 38, 39] were sequenced in multiple sequencing runs (S1 Table). In a whole genome alignment of all 121 *L*. *monocytogenes* genomes, a total 166,603 core-genome SNPs were identified. Phylogenetic inference based on these SNPs identified three *L*. *monocytogenes* lineages among the isolates (Fig 1). Within lineage I genomes (n = 62) there were a total of 23,860 core SNPs identified, while 82,970 core SNPs were identified among the lineage II genomes (n = 56), and 27,405 core genome SNPs were identified among the three lineage III genomes (n = 3). Lineage III genomes had an average of 69,645 and 93,791 SNP differences with lineages I and II genomes, respectively, while there was an average of 77,978 SNP differences between lineage I and lineage II genomes. There were on average 18,333 SNP differences between lineage III genomes, 15,074 SNP differences between lineage II genomes, and 5,684 SNP differences between lineage I genomes.

The phylogenetic analysis was consistent with serogrouping results as all 1/2b and most 4b isolates clustered within lineage I (Fig 1). Within this lineage, 1/2b isolates (n = 37) and 4b isolates (n = 24) formed separate clades (Fig 1). Two isolates identified by PCR as serogroup 4b were identified as members of lineage III (S1 Table). We were not able to determine the genomic serogroup of four PCR serogroup 4b isolates, but these were all members of lineage I and clustered among the other serogroup 4b isolates; thus, the PCR-based serogrouping results were consistent with their phylogenetic placement (S1 Table) (Fig 1). Four lineage I isolates were determined to be serogroup IVb-v1 by genomic analysis. All PCR serogroup 1/2b isolates were confirmed to be serogroup IIb by genomic analysis. Consistent with other studies, all PCR serogroup 1/2a isolates (n = 55) were members of lineage II which, based on the phylogenetic analysis, consisted only of 1/2a isolates and one isolate for which a serogroup could not be determined (Fig 1). This isolate, ARS-CC9282 (isolated from a bulk tank milk filter from CA in 2014), was deeply rooted in an ancestral node of lineage II, distant from all other genomes analyzed in this study. There were two PCR serogroup 1/2a isolates for which a serogroup could not be determined by a genomic analysis. As stated earlier, two PCR serogroup 4b isolates were determined to be lineage III isolates which also included one isolate for which a PCR serogroup could not be determined. Two lineage III isolates were identified as serogroup L(4) and one was identified as serogroup L(6) by genomic analysis.

**Fig 2:**
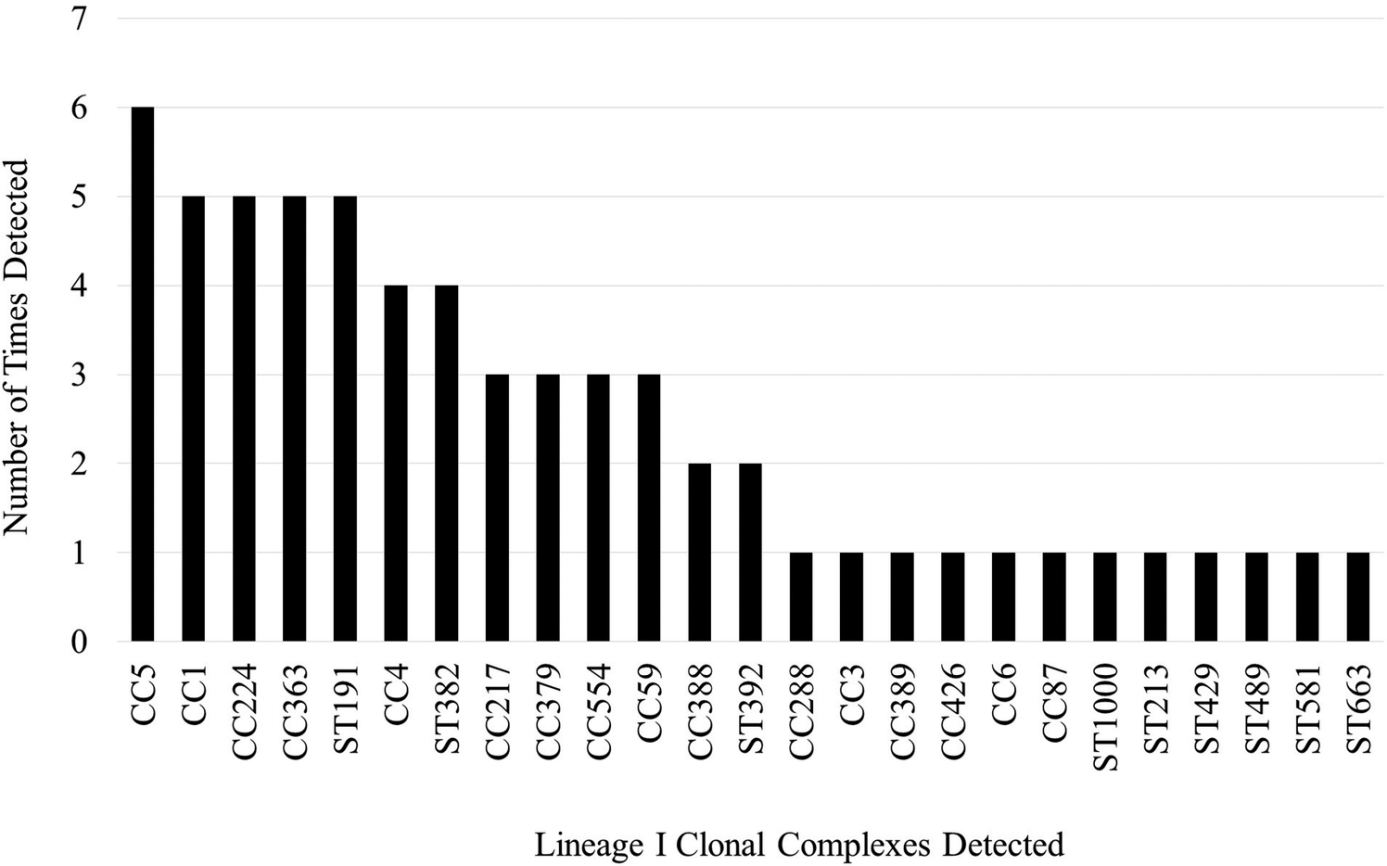
Frequency of detection for all lineage I clonal complexes.

There was a total of 60 sequence types (ST) belonging to 56 clonal complexes (CC) and 59 virulence types (VT) within this set of 121 *L*. *monocytogenes* (Tables 1 and S1). Within lineage I there were 25 CCs consisting of 28 STs (Fig 2 and Tables 1 and S1). Within this lineage, the most frequently isolated CCs were CC5 (6 isolates, 9%), CC1, CC224, CC363, and singleton ST191 (5 isolates each, 8% each). Four isolates (6% of lineage I) were novel STs (two ST999, one ST1000, and one ST1001). Within this lineage the most common VTs were VT63 (ECVI) (6 isolates, 9%), and VT124, VT127 and VT108 (5 isolates each). VT63 (ECVI), VT124, VT127, and VT108 correspond to CC5, CC224, ST191, and CC363, respectively. Seventeen isolates (27% of lineage I) were identified as 10 novel VTs (VT127, VT128, VT129, VT130, VT131, VT132, VT133, VT135, VT136, VT137). Thirty percent of the lineage I strains were identified as epidemic clones (EC) by a MVLST analysis (Table 1).

**Table 1:** Numbers of isolates, states from which these isolates were recovered, sequence types, clonal complexes (including singletons), virulence types, and epidemic clones (VT epidemic clones) for each lineage represented in this study.

Lineage	No. of Isolates	No. of States	No. of Sequence Types (ST)	No. of Clonal Complexes (CC)	No. of Virulence Types (VT)	No. of Epidemic Clone (EC) Isolates (% of Total)
Lineage I	62	17	28	25	25	19 (30%)
Lineage II	56	12	29	28	31	12 (21%)
Lineage III	3	3	3	3	3	0
Total	121	19	60	56	59	31 (25%)

**Fig 3:**
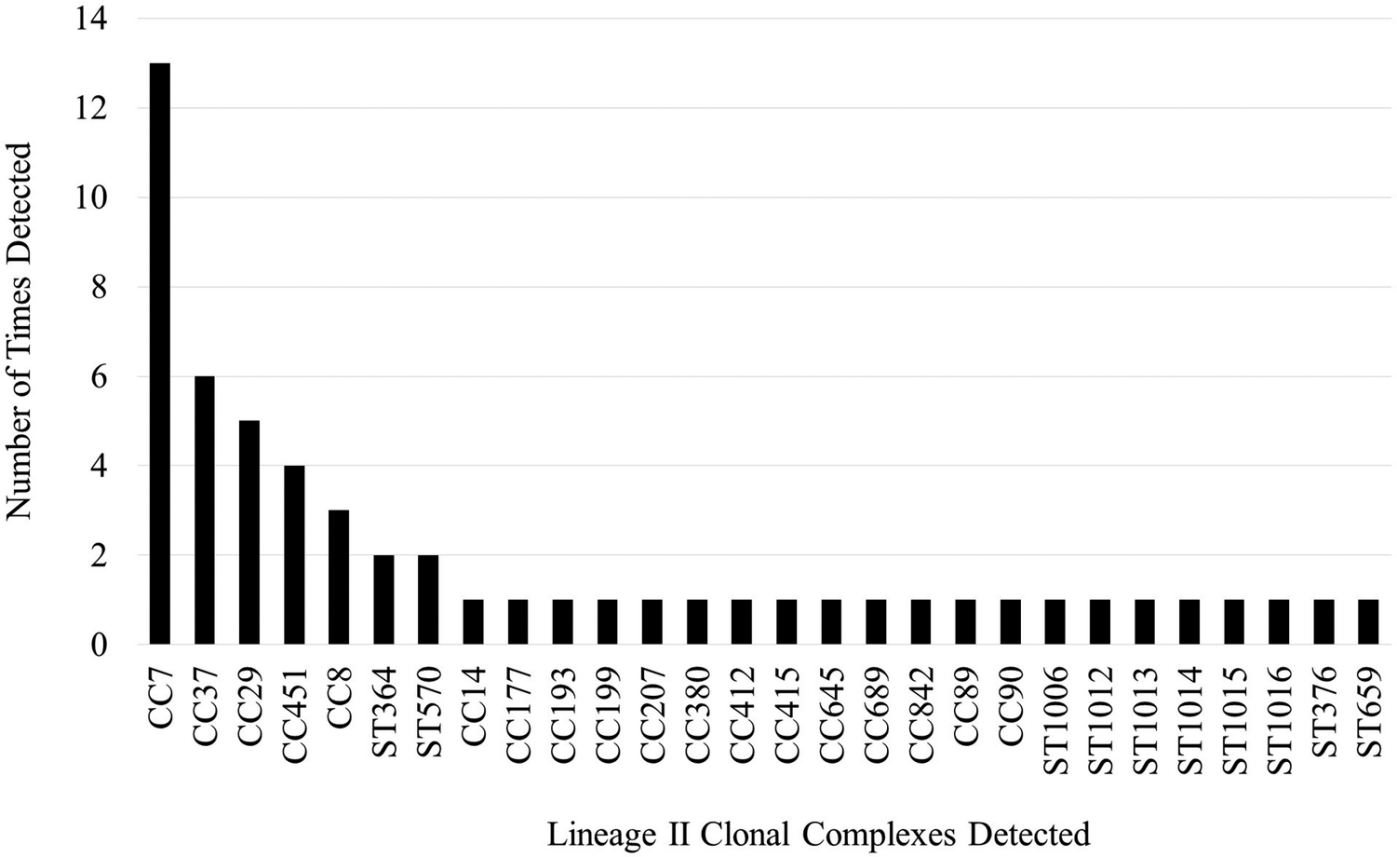
Frequency of detection for all lineage II clonal complexes.

Within lineage II there were 28 CCs consisting of 29 STs (Fig 3) (Table 1) (S1 Table). The most common CCs were CC7 (13 isolates, 23% of lineage II isolates), CC37 (6 isolates, 10%), and CC29 (5 isolates, 9%). There were eight isolates (14% of lineage II) that were assigned to eight novel STs (ST-1004, ST-1006, ST-1007, ST-1012, ST-1013, ST-1014, ST-1015, ST-1016). The most common VTs were VT56 (ECVII) (11 isolates, 19%), VT61 (6 isolates, 10%), VT74 (5 isolates, 8%). VT56 corresponds to a subset of CC7 isolates, while VT61 and VT74 correspond to CC37 and CC29, respectively. Twenty-five isolates (44% of lineage II) were identified as 19 novel VTs (VT139 to VT144, and VT147 to VT159). Twenty-one percent of the lineage II strains were identified as epidemic clones.

Only three lineage III isolates were identified in this study and each of these were novel STs (ST-998, ST-1002, and ST-1003). They were also novel VTs (VT138, VT145*, and VT146*). VT145* and VT146* were both lacking the *inlC* (internalin C) virulence gene, which is used as a marker for the MVLST scheme.

Within each lineage, several CCs were isolated over a broader geographic and temporal scale than others. Within lineage II, CC7 isolates represented five states (CA, CO, MI, WI, MN) in 2002, 2007, and 2014. CC37 was isolated from four states (MI, MN, NY, PA) in 2002, 2007, and 2014.

Within lineage I, CC5, which includes VT63 (ECVI), was detected in four states (CA, NY, IN, MI), but only represented 2002 and 2007. CC224 was isolated from four states in 2002 and 2007. CC1 was isolated from three states (NY, IA, MI) in 2005, 2007, and 2014. CC363 was isolated from three states and in 2002, 2007, and 2014. ST191 was isolated in four states but only in 2002.

Within lineage I the most frequently detected CC in 2002 was singleton ST191, while CC1, CC5, CC224, and CC363 were the most frequently detected in 2007, and none were more frequently detected than others in 2014. Within lineage II, CC7 was the most frequently detected CC in 2002, 2007, and 2014. CC37 was the second most frequently detected in 2002 and 2007.

**Fig 4:**
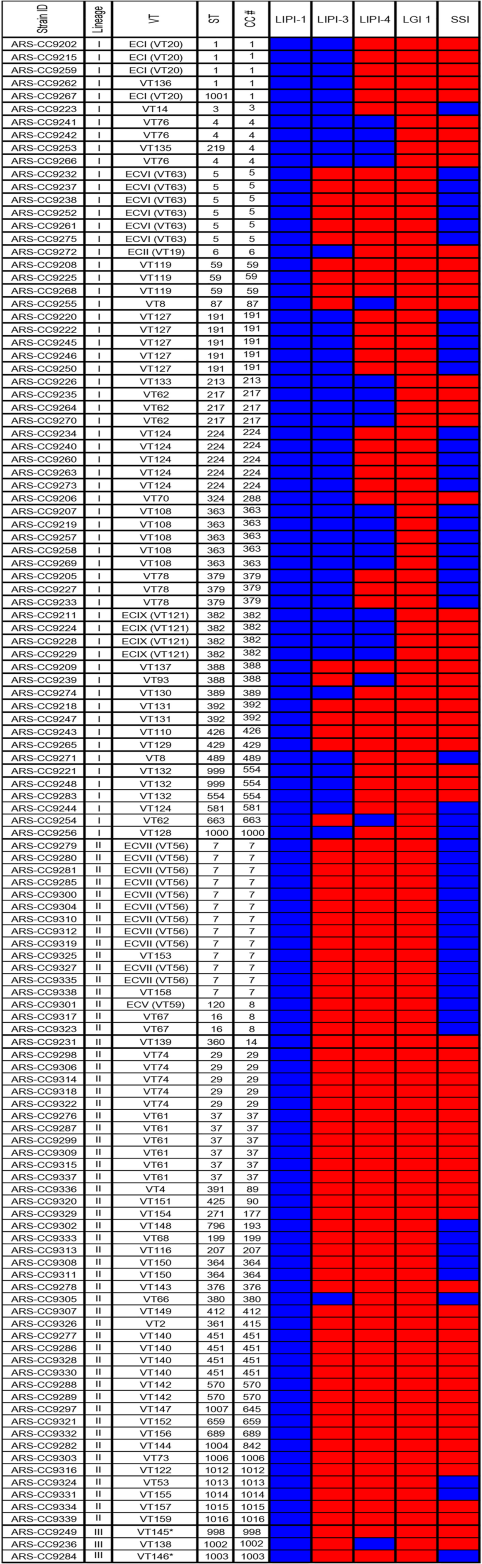
Presence/absence of genomic island and pathogenicity islands identified among the study CCs. Blue = detected. Red = not detected.

**Fig 5:**
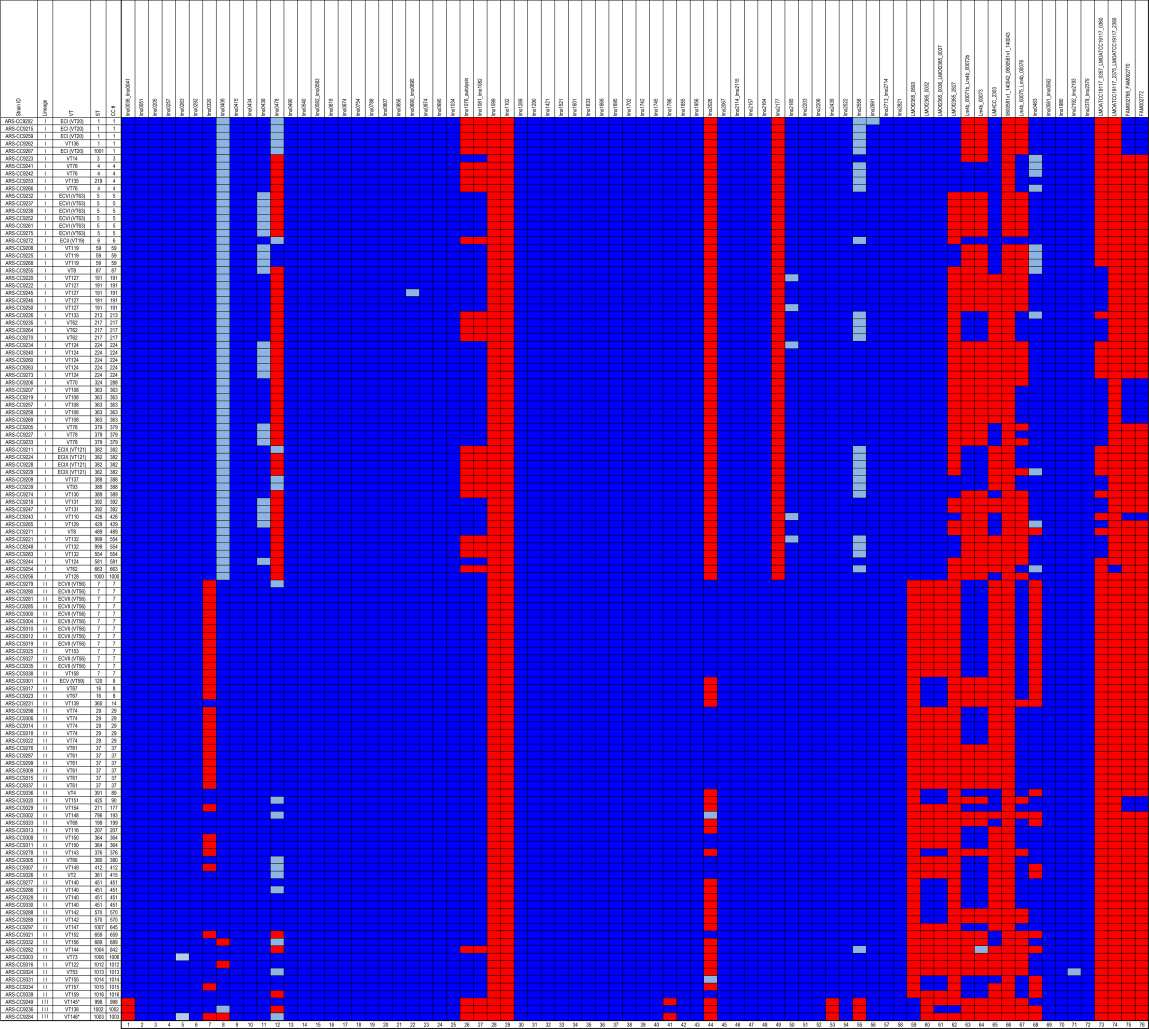
Presence/absence of genes associated with virulence among the study isolates. Blue = detected. Light blue = shorter than the reference gene used in the analysis. Red = not detected. Columns 1 to 62 (at the bottom of the figure) were based on the analysis described by Kuenne et al. [47] and columns 63 to 76 were based on the analysis described by Maury et al. [9].

The presence of core genome virulence factors, pathogenicity islands, and genomic islands was evaluated for all isolates in this study and the results indicated a high level of variability in their presence/absence and sequence similarity (Figs 4 and 5). None of the genomes encoded the *Listeria* Genomic Island-1 (LGI-1). There were also no insertions identified at the LGI-1 locus in any of the genomes (lmo1702/lmo1703) indicating this locus was empty for all isolates. As expected, no genomes encoded the *Listeria* Pathogenicity Island-2 (LIPI-2). *Listeria* Pathogenicity Island-1 (LIPI-1) was identified in all 121 *L*. *monocytogenes* genomes. However, the *inlA* gene, known to be one of the major virulence factors of *L*. *monocytogenes*, was truncated in three genomes due to premature stop codons [ARS-CC9308 (ST364), ARS-CC9310 (CC7), ARS-CC9232 (CC5)] and was shorter in seven genomes due to an internal 9-bp deletion.

*Listeria* Pathogenicity Island-3 (LIPI-3) was more frequently detected in lineage I (72% of isolates and 68% of CCs) than in lineage II (1 isolate with ca. 96% similarity) or lineage III (0 isolates) (Fig 4 and Table 2). LIPI-3 was identified in 17 different CCs and was identified in each genome within these CCs within lineage I. All of these isolates encoded a homologous LIPI-3 region with at least 98.8% nucleotide sequence similarity to the canonical LIPI-3 sequence. A region with 96% nucleotide sequence similarity to the canonical LIPI-3 was identified in the lineage II genome ARS-CC9305 (CC380). *Listeria* Pathogenicity Island-4 (LIPI-4) was identified more frequently in lineage I (32% of isolates and 28% of CCs) than in lineage II (0 isolates) (Fig 4 and Table 2) and was also identified in a single lineage III isolate (ARS-CC9236). This island was found in 8 CCs/singletons. The stress-survival islet (SSI) was identified in 46% of lineage I genomes, 43% of lineage II genomes and a single lineage III genome (Fig 4).

**Table 2:** Virulence factors that were more frequently detected in one lineage than the other (lineages I and II).

Gene/Locus	Annotation	% Present in Lineage I Isolates (% Present in CC)	% Present in Lineage II Isolates (% Present in CC)	P-Value
lmo0320 ^(^^a^^)^	Peptidoglycan bound surface protein ^(^^b^^)^	100 (100) ^(^^c^^)^	39 (61) ^(^^c^^)^	< 0.001 ^(^^d^^)^
lmo0478	Secreted protein	19 (20)	94 (89)	< 0.0001
lmo1076	Autolysin (aut)	60 (60)	98 (96)	< 0.01
lmo1081 to lmo1082	Glucose-1-phosphate thymidyl transferase / dTDP-sugar epismerase	60 (60)	98 (96)	< 0.01
lmo2026	Peptidoglycan binding protein	0 (0)	58 (39)	< 0.001
lmo2177	Hypothetical protein	0 (0)	100 (100)	< 0.0001
LMOf2365_0693	Cell wall surface anchor family protein	100 (100)	0 (0)	< 0.0001
LMOf2365_0032, LMOf2365_0036 to LMOf2365_0037	Arginine/Ornithine antiporter	100 (100)	26 (32)	< 0.0001
LMOf2365_2627	ABC Transporter	32 (32)	0 (0)	< 0.01
Lm4b_00073	Hypothetical protein	24 (24)	60 (50)	< 0.05
lmo0463	Peptidoglycan bound protein (LPXTG motif)	98 (96)	51 (54)	< 0.001
LMOATCC19117_0357 to LMOATCC19117_0360	Epismerase/dehydrogatase family protein and transcriptional regulator	41 (40)	0 (0)	< 0.001
LIPI-3	Listeria Pathogenicity Island 3	72 (68)	1 (3)	< 0.0001
LIPI-4	Listeria Pathogenicity Island 4	32 (28)	0 (0)	< 0.01

(a) Virulence factor loci

(b) Virulence factor annotations

(c) Frequency of detection in isolates (and CCs).

(d) Fisher’s exact test P-value. A P-value < 0.5 indicates a significant difference in the detection frequency of that virulence factor between lineages I and II.

The presence of additional genomic regions previously identified as being associated with virulence in *L*. *monocytogenes* was evaluated in the study genomes (Fig 5). At least 49 of these regions were identified in every genome and none of the genomes encoded all regions. Lineage-specific trends in the presence/absence of virulence regions and gene length were observed. Three regions, lmo0036 to lmo0041, lmo2439, and lmo2558, were not detected in all lineage III genomes. lmo0409 (internalin) was shorter than the reference gene from *L*. *monocytogenes* EGD-e in all lineage I isolates, and was not detected in two lineage II genomes and one lineage III genome. lmo2558 (autolysin, amidase) was shorter than the reference gene in 40% of lineage I genomes and only one lineage II genome. Based on a Fisher’s exact test 14 putative virulence regions, including LIPI-3 and LIPI-4, were more frequently identified in CCs of one lineage than the other (lineage I or II) (P < 0.05) (Table 2).

Acquired antibiotic resistance genes were detected *in silico* in only four genomes. Tetracycline resistance ribosomal protection protein gene *tet(M)* was detected in four genomes (ARS-CC9268, ARS-CC9333, ARS-CC9320, and ARS-CC9280), and *tet(S)* was detected in the ARS-CC9268 genome. Macrolide resistance gene *erm(B)* was also detected in the ARS-CC9268 genome. In a microdilution assay that tested for sensitivity to a panel of antibiotics ARS-CC9333, ARS-CC9320, and ARS-CC9280 were resistant to tetracycline, and ARS-CC9268 was resistant to tetracycline and erythromycin.

## Discussion

*L*. *monocytogenes* is occasionally isolated from unpasteurized dairy cow milk, dairy products such as soft cheeses, pasteurized milk that has been contaminated, feces of both asymptomatic dairy cows and cows with listeriosis, and dairy farm environments [16, 17, 24, 31–38, 52–54]. In recent years, several listeriosis outbreaks have been linked to dairy products including unpasteurized milk [26, 55, 56]. Although, dairy products have been repeatedly shown to occasionally harbor this pathogen, the diversity of *L*. *monocytogenes* strains isolated from unpasteurized milk and dairy farms remains underdescribed. Results of this analysis demonstrate that there is a high level of within-lineage diversity, with respect to virulence types, sequence types, clonal complexes, and the distribution of virulence factors among isolates collected from BTM and BTMF. Our analysis clearly indicates that within-lineage diversity is high and multiple subtypes of *L*. *monocytogenes* are present in dairy production systems. These results are consistent with those of other studies that have investigated the diversity of *L*. *monocytogenes* in fecal and environmental samples collected from dairy farms indicating that bovine feces, environmental dairy farm samples, and unpasteurized milk can harbor a diverse suite of strains [16, 17, 24, 31–38, 52–54]. However, it should be noted that during these studies the number of total dairy operations from which *L*. *monocytogenes* was isolated was relatively low [16, 17, 19].

Previous studies targeting specific environments or food products (non-dairy/non-dairy farm) have often identified a similar or lower level of strain diversity (at the CC, ST, or VT level of resolution) suggesting bulk tank milk collected across the United States may harbor a greater diversity of *L*. *monocytogenes* than some other food products or environments. For example, Martín et al. [12] identified 17 ST among 109 isolates from meat processing plants with only 8 STs being isolated more than once. Wang et al. [13] identified 11 STs, while Wu et al. [11] identified 14 STs in two separate studies of ready-to-eat (RTE) meats in China. *L*. *monocytogenes* is typically introduced to BTM through fecal contamination during the milking process [57] and cows shedding *L*. *monocytogenes* can become infected through the consumption of contaminated silage, water, other environmental materials, wildlife, and/or from exposures to the feces of other cows that are shedding this organism. Thus, the multitude of potential routes of *L*. *monocytogenes* transmission within dairy herds may ultimately result in the high level of strain diversity observed in this study. To-date, no definitive conclusions have been made concerning the pressures that influence the proliferation of certain *L*. *monocytogenes* strains in the dairy farm environment and multiple studies have demonstrated high serogroup-level diversity in dairy animals and unpasteurized dairy [16, 17, 19, 38].

Although we observed a relatively high level of diversity among the *L*. *monocytogenes* isolates in this study, our analysis also demonstrated within-lineage predominance of several CCs. Within lineage II, CC7 and CC37 were the predominant CCs. Within lineage I there did not appear to be a predominance of a single CC, but rather a similar proportion of several CCs (CC5, CC1, CC224, CC363, and ST191). However, only CC363 of lineage I was isolated during each survey year, while CC7 and CC37 of lineage II were isolated during each survey year. The repeated isolation of CC7 and CC37 over time and from multiple states provides evidence of their predominance among the *L*. *monocytogenes* that occasionally contaminate BTM. CC7 isolates have been recovered globally (North and South America, Europe, Oceania, Africa, and Asia) from a variety of sources such as wild animals, ruminants, poultry, silage, fish, slaughterhouse floors, compost, and human CNS and MN infections indicating it may be capable of persisting in many environments [44]. However, CC37 represents a relatively rare group of strains that have been isolated from urban and rural environments as well as from farms in North America and dairy and meat products in Europe. Presence of these strains in dairy products and farm environments suggests CC37 may be selected for in the dairy farm environment. A study by Linke et al. [58] identified CC37 as one of the most frequently isolated groups of strains from soils in proximity to wild and domestic ruminants. Multiple isolates were also recovered from non-animal host environments including a forested region identified as “pristine” which also indicates CC37 may persist for long periods outside of the animal host. Similarly, Dreyer et al. [33] frequently identified CC37 isolates (ST37) in both ruminant fecal samples and environmental samples collected at or near locations of ruminant farms. Along with CC37, CC7 isolates were recovered during all survey years from five of the states tested indicating that among the *L*. *monocytogenes* isolated from BTM and BTMF, they are the predominant CCs. These results indicate that either these CCs are relatively successful in many environments, including dairy operations, or they are selected for on the dairy farm, in the milking process, and/or have the potential to proliferate in unpasteurized milk. Recent studies have demonstrated that biofilm formation/persisting abilities are not equal among strains and the potential of strains to form biofilms in milking equipment may influence the predominance of some strains in BTM/BTMF [27, 59]. CC/ST predominance has been identified in other studies, indicating that certain groups of strains may be adapted for persistence in specific environments [11–14]. Further studies on the persistence of representative isolates of predominant CCs in dairy animals, unpasteurized milk, and their ability to form biofilms in milking equipment may help identify causal factors of strain predominance.

Historically all strains of *L*. *monocytogenes* have been considered to be food adulterants and pathogenic to humans. However, recent studies indicated that some strains are more strongly associated with severity or type of illness and/or frequency of infections [9, 14, 33]. Several isolates recovered in this study had characteristics of strains known to be strongly associated with CNS and MN infections. Of particular concern is the isolation of isolates within several CCs associated with severe clinical infections, particularly CC1, CC4, and CC6, which have been shown to be strongly associated with human listeriosis in a comparison of clinical CCs to food-isolated CCs [9]. These complexes represent 8% of the total isolates and 26% of the states from which *L*. *monocytogenes* isolates were collected. Further, 25% of the total isolates were identified as epidemic clones by MVLST analysis (30% of lineage I and 21% of lineage II) indicating that they are highly similar to strains that are frequently recovered from listeriosis cases and outbreaks that are temporally and geographically unrelated. These results indicate that a significant proportion of the isolates that are recovered in BTM in the United States are genomically similar to those that are relatively frequently isolated from human clinical cases.

At a more discriminatory level, we identified multiple genomic regions known to contribute to *L*. *monocytogenes* virulence that were either consistently present in all or most isolates, or sporadically distributed among isolates and apparently enriched in specific CCs or lineages. Most *L*. *monocytogenes* virulence-associated genes are encoded in the core genome as is evident in Fig 5 [47, 60–63]. The *inlA* gene is known to be a major virulence factor of *L*. *monocytogenes* and truncations due to premature stop codons are responsible for significant attenuation of virulence. Only three of the dairy isolates had truncated *inlA* genes, while seven isolates had 9 bp internal deletions. Other major virulence factors were detected among the study set and the unequal distribution of some virulence factors indicates that the virulence potential among the BTM/BTMF isolates may vary considerably. These results also demonstrate that several strains encoding pathogenicity islands that are potentially involved in severe disease outcomes were repeatedly detected among the isolates recovered from BTM. For example, 68% of lineage I CCs encoded LIPI-3. This island is also known as the listeriolysin S cluster and it encodes a haemolysin that was thought to contribute to survival in polymorphonuclear neutrophils [64]. LIPI-3 has since been shown to act as bacteriocin that acts to target the host gut microbiota [65]. Another virulence factor, LIPI-4, implicated in placental infections [9], was identified in 21 isolates (17% of all isolates, 32% of lineage I isolates, 1 lineage III isolate, and 0 lineage II isolates). Until recently, LIPI-4 has only been identified in CC4 isolates, and results of this study indicate it is present in several other lineage I CCs as well as in a single lineage III CC. The repeated presence of this pathogenicity island among isolates recovered from unpasteurized milk underscores the potential risks of consumption of raw milk, especially for certain members of the population, including pregnant women.

## Conclusions

This study established that *L*. *monocytogenes* isolated from dairy farm samples (BTM and BTMF) across the United States is highly heterogeneous, and that there is evidence of a predominance of certain clonal complexes among lineage II isolates (CC7 and CC37). The results of our analysis also indicated that a significant portion of isolates recovered from BTM/BTMF are the same CCs and VTs as those that are frequently isolated from human clinical cases and outbreaks on a global scale, and that many of those strains encode virulence factors implicated in severe disease (LIPI-3 and LIPI-4). These findings contribute to a better understanding of the genomic diversity and virulence profiles of *L*. *monocytogenes* present in unpasteurized BTM in the United States. Future work should be conducted to evaluate the mechanism of persistence of predominant strains in environments and materials found on dairy farms and in BTM.

## Supporting information

S1 Table Lineage, serogroup, sequence type (ST), clonal complex (CC), virulence type (VT) and accession numbers of *L*. *monocytogenes* isolates from BTM, BTMF, and milking equipment. A = serogroup as determined by Doumith et al [40]. B = Serogroup as determined by genome analysis [44].
(XLSX)
